# Sensitivity Enhancement of a Concave Shaped Optical Fiber Refractive Index Sensor Covered with Multiple Au Nanowires

**DOI:** 10.3390/s19194210

**Published:** 2019-09-27

**Authors:** A. K. Pathak, B. M. A. Rahman, V. K. Singh, S. Kumari

**Affiliations:** 1 Optical Fiber Laboratory, Indian Institute of Technology (Indian School of Mines), Dhanbad 826004, India; 2 Department of School of Mathematics, Computer Science and Engineering, City University London, London EC1V 0HB, UK; 3 Department of Electrical Engineering, Indian Institute of Technology, Patna 801106, India

**Keywords:** refractive index, surface plasmon resonance, nanowires, microfluidic channel, sensitivity

## Abstract

In the present paper, a new kind of concave shaped refractive index sensor (CSRIS) exploiting localized surface plasmon resonance (LSPR) is proposed and numerically optimized. The LSPR effect between polaritons and the core guided mode of designed CSRIS is used to enhance the sensing performance. The sensor is characterized for two types of sensing structures coated with gold (Au) film and Au nanowires (AuNWs), respectively. The influence of structural parameters such as the distance (D) of the concave shaped channel (CSC) from the core, the diameter of the nanowire (d_n_) and the size (s) of the CSC are investigated here. In comparison to Au film, the AuNWs are shown to significantly enhance the sensitivity and the performance of the designed sensor. An enhanced sensitivity of 4471 nm/RIU (refractive index unit) is obtained with AuNWs, for a wide range of analytes refractive index (n_a_) varying between 1.33 to 1.38. However, for conventional Au film; the sensitivity of 808.57 nm/RIU is obtained for the same range of analytes.

## 1. Introduction

Surface plasmon resonance (SPR) is the resonant oscillation of the conduction electrons at the metal–dielectric interface, which is stimulated by the incident light [1]. The SPR phenomenon can be classified into two categories, namely SPR and localized surface plasmon resonance (LSPR). The matching of wave vector of incident wave with the surface plasmon (SP) leads to the popularly known phenomenon of SPR at the metal–dielectric interface [2,3], while the LSPR phenomenon refers to the collective oscillations of the electron charge confined around the metallic nanostructures and demonstrate enhanced near-field amplitude at the resonance wavelength [4]. The first experiment based on the SPR phenomenon for bio-sensing and gas detection was conducted by Liedberg et al. [5]. Following that, SPR has attained significant attention due to its sensitive performance toward its surrounding media. In the traditional SPR configuration reported by Kretschmann, a prism with a thin metal deposited on its base was used, and the SPs were excited on the metal–dielectric interface in the presence of p polarized light, at a certain angle. However, the prism based free space SPR configuration is bulky in size and may not be suitable for remote sensing, which limits its application and large-scale fabrication for real monitoring.

To overcome these drawbacks, the feasibility of SPR based optical fiber devices in various sensing applications is explored, in order to achieve enhanced sensitivity. However, some optical fiber sensors require the complete removal of cladding so as to enhance the sensitivity which leads to a fragile sensor and experimental complexity [6]. The first SPR based fiber sensor was developed and demonstrated by Jorgenson in 1993, in which a thin gold film was used to coat the fiber core to excite the SPR. Since then, a large number of SPR based fiber sensors have been reported, e.g., fiber Bragg grating (FBG), tapered fiber structure, photonic crystal fiber, D-shaped polished fiber, etc. [7,8,9]. Out of these reported sensors, the sensors developed by using the D-shaped polished fiber became more attractive among researchers due to their easy fabrication, high sensitivity, less fragile structure and the possibility to be used in any hazardous media. In the earlier reported articles, Au and silver (Ag) metals were used widely in various SPR based devices due to their strong absorption property. In comparison to Au, Ag exhibits a sharper resonance peak, and provides a high detection accuracy, but Ag can be easily oxidized [10] in air, and hence researchers have found Au to be the most promising metal for sensing. After the wide use of thin film, the sensor based on metallic nanowire has attracted significant attention from the scientific community as being a good plasmonic medium, in the development of sensing devices for various applications. In such sensors, the formation of plasmonic modes takes place around the nanowire [11] and the resonance peak is excited when the dielectric core mode is coupled with the plasmonic modes at a particular wavelength, which is known as the resonance wavelength. Various sensors have been developed with such metallic nanowires, using LSPR technology, but this has mostly been for temperature sensing [12,13]. While the reported sensors achieved a good sensitivity in terms of temperature sensing, the main challenges of complexity in fabrication, and filling the hole with selective analytes and the nanowire, still remain.

In the present paper, we report a new kind of LSPR based optical fiber refractive index (RI) sensor using CSC. This sensor is designed and analyzed using the finite element method (FEM) technique based on COMSOL Multiphysics. The work illustrates the comparative analysis of CSRIS in which we have designed two types of sensors, one covered with Au thin film and the other covered with multiple AuNWs. The designed sensor is based on the wavelength interrogation technique which uses the modal loss to optimize its structural parameters and systematically study sensing response. The simulation results show that the sensitivity of the designed sensor can be greatly enhanced by covering the CSC with multiple AuNWs instead of Au film. The sensor achieves both high wavelength and amplitude sensitivity of 4471 nm/RIU and 214 RIU^−1^ respectively with AuNWs for n_a_ varying from 1.33 to 1.38. Various chemicals such as acetone, silicone oil, ethanol, polylactic acid, toluene, and glucose have a RI that lies between 1.33–1.46 [14,15,16], and hence, we use the designed sensor to measure the RI of similar analytes.

## 2. Design and Theoretical Modelling

**Figure 1:**
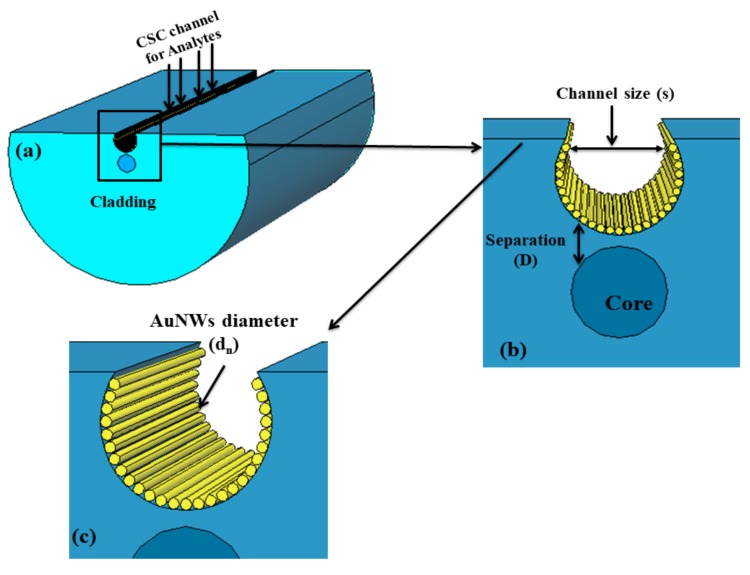
Schematic diagram of the designed concave shaped refractive index sensor (CSRIS) (**a**) cross section of designed sensor, (**b**) and (**c**) are the zoomed in and further magnified diagram of gold nanowires (AuNWs) covered concave shaped channel (CSC), respectively.

Figure 1 illustrates the schematic cross-sectional view of the designed CSRIS. AuNWs are arranged along the length of the fiber inside the CSC. In order to maintain the symmetry of nanowires (NWs) on the boundary of CSC, the diameter of NWs is varied in natural numbers. The proposed sensor is designed by using a standard single-mode fiber with core/cladding diameter of 9/125 µm. The distance of CSC from the core, the diameter of the AuNWs and the size of the CSC are defined by D, d_n_, and s, respectively. The most versatile stack-and-draw method and lateral polishing technology can be used to fabricate the D shape fiber [17]. Later, the concave shaped can be fabricated on this D shape fiber by focusing the ultrashort pulse on the polished surface transversally. According to a previously reported article, a microchannel can be easily fabricated at the polished D shaped edge near the core of optical fiber by considering a Gaussian field distribution. The Gaussian field distribution is positioned at the edge of polished surface and part of the area exposed to the field is removed to form the sensing structure, as shown in Figure 1. The Gaussian field profile can be written in the form of the following equation [18]:

(1) $$y=y_{0}+{\mathrm{Ae}}^{-\frac{{\left(x-x_{c}\right)}^{2}}{2\omega^{2}}}$$

where y_0_, x_c_, ω and A represents offset, center, width, and amplitude, respectively. Later the nanowire can be deposited by using colloidal solution of Au inside the CSC.

In the designed sensor, the core and cladding of the used fiber are made of GeO_2_ doped silica and fused silica respectively, the RI of which is obtained by using the Sellmeier equation and can be calculated by the following:

(2) $$n^{2}\left(\lambda^{2}\right)\;=1+\;\frac{B_{1}\lambda^{2}}{\lambda^{2}-C_{1}}\;+\;\frac{B_{2}\lambda^{2}}{\lambda^{2}-C_{2}}\;+\;\frac{B_{3}\lambda^{2}}{\lambda^{2}-C_{3}}$$

where, B_1,2,3_ and C_1,2,3_, are Sellimeiers coefficients, the values of which are taken from a previously reported article [19]. The material dispersion of Ag nanowire is calculated by the Lorentz–Drude model, as it gives more accurate prediction of permittivity and is closer to the experimental values, and this can be defined as [20]:

(3) $$\varepsilon_{m}=1-\frac{\Omega_{p}^{2}}{\omega \left(\omega -{i\Gamma }_{0}\right)}+\sum_{j=1}^{k}\frac{f_{j}\omega_{p}^{2}}{(\omega_{j}^{2}-\omega^{2})+{j\omega \Gamma }_{i}}$$

where ε_m_ and ω_p_ represent the dielectric constant and the plasma frequency respectively, whereas k is the number of oscillators with frequency ω_p_ and strength f_j_. Ω_p_ = √(f_0_.ω_p_), shows the plasma frequency related with intraband transition. The proposed sensor is designed and characterized using COMSOL Multiphysics based on FEM [21,22]. The width of this CSC is in the order of microns and hence this type of sensing structure requires a very small amount of analyte for detection. Here in the proposed design we have considered ‘extremely fine’ meshing for AuNWs, while for the whole structure, ‘finer’ meshing is used. Furthermore, the whole cross section of the designed sensor is divided into various triangular subdomains. In total, 271962 mesh elements are used in the analysis.

## 3. Results and Discussion

**Figure 2:**
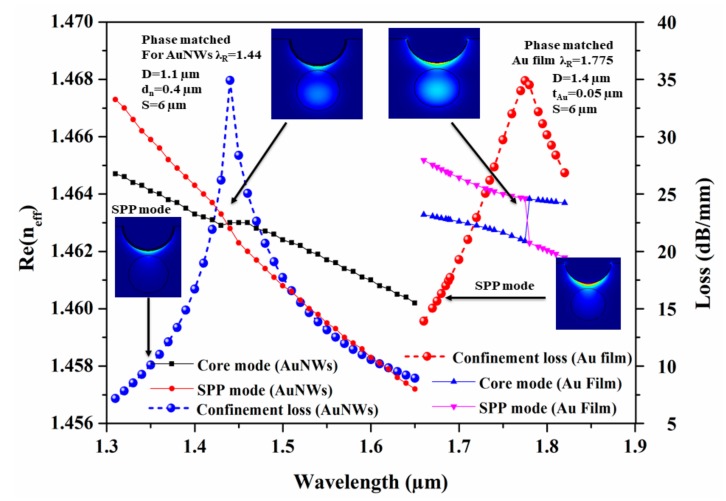
Dispersion curve for both Au thin film/NWs.

The proposed CSRIS is based on the LSPR phenomenon that takes place between the surface plasmon polaritons (SPP) and the core guided modes. It occurred due to the interaction of enhanced evanescent waves with target analytes of varying RI (n_a_). These enhanced evanescent waves easily excite the conduction electron on Au film/NWs at a certain wavelength, which is known as the resonance wavelength (λ_R_). At this λ_R_, these conduction electrons of Au film and AuNWs generate the dielectric–metal interface guided plasmon waves. Figure 2 illustrates the dispersion curve of designed sensor for both Au NWs/film. The plot shows the variation of real effective refractive index (Re(n_eff_)) of SPP and core guided modes at different wavelengths along with the modal loss spectra for both thin film and NWs. The plots are shown by blue and red circles for AuNWs and Au film, respectively. In the plot, the Re(n_eff_) of highly dispersive SPP mode for both AuNWs and Au film, as shown by red circles and pink triangles, decrease linearly, while their core guided modes, shown by black squares and blue triangles, remain almost constant throughout the variation. Figures shown as insets are the electric field distribution for phase matched and SPP modes for both AuNWs and Au film when n_a_ = 1.33 with their optimized parameters. In the inset images, we can clearly see that in both cases (i.e., for Au NWs/film) the maximum portion of the light is confined in the fiber core. However, with increasing wavelength, most of the electromagnetic field extended into the cladding region and resulted in the reduction of Re(n_eff_). The confinement loss for both Au NWs/film of the CSRIS can be calculated by the following equation [23]:

α(dB/m) = 8.686. k_0_. Im (n_eff_) (4)

where the wavenumber (k_0_) is defined as k_0_ = 2π/λ. Generally, the plasmonic modes demonstrate more loss compared to the dielectric core guided modes. Hence, when these two modes get mixed with each other near the λ_R_, the loss value of the core mode increases sharply, as shown by the blue and red circles for both AuNWs and Au film, respectively.

**Figure 3:**
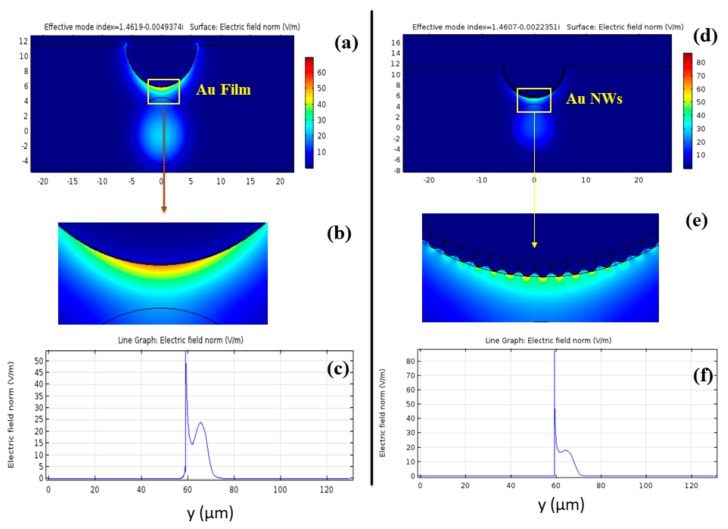
(**a**) and (**d**) show electric field distribution E_y_ near film and NWs, respectively, (**b**) and (**e**) show the enlarged view; (**c**) and (**f**) show the E_y_ field variation along the y-axis, for Au film and NWs, respectively.

The electric field E_y_ distributions at n_a_ = 1.33 for Au film and AuNWs at their resonance are shown in Figure 3a,d respectively. Figure 3b,e illustrate the enlarged view of the fields near the metals. The used optimized parameters for both Au film/NWs are shown in Table 1.

Figure 3b,e illustrate the magnified image of mode coupling of dielectric core guided mode with the SPP mode for Au film and AuNWs respectively. The moderate E_y_ field value in the core region is clearly visible, but a more localized E_y_ field around the Au film and AuNWs is also observed. To get a more intuitive view of the local plasma enhancement, we have shown the normalized field profiles E_y_ in Figure 3c,f for Au film and AuNWs respectively along y-axis. Here we can observe from both figures that the peak value for AuNWs is higher in comparison to Au film i.e., the normalized electric field strength for AuNWs is higher. Hence, it demonstrates that more energy from the dielectric core mode is coupled into the SPP modes of AuNWs, and therefore it can be more sensitive to the surrounding media than Au film.

**Figure 4:**
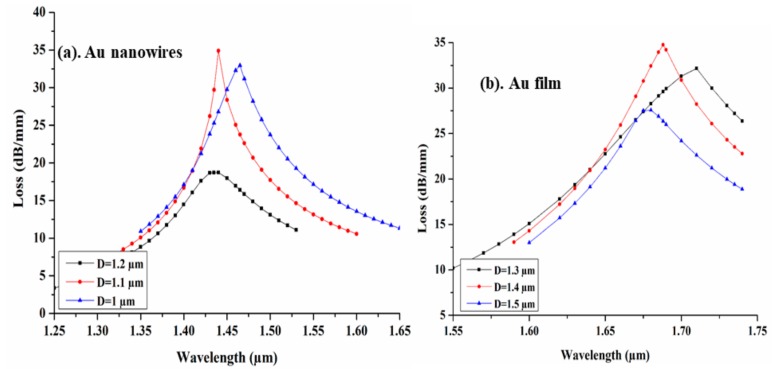
Variation in confinement loss spectra at various separations between CSC and the core for (**a**) AuNWs and (**b**) Au film.

During the analyses, we have considered that light propagates along the Z-direction, while all the modal analysis is performed in the XY plane. The distance of the CSC from the core is of critical sensing performance, since it helps to determine the strength of evanescent waves and thus influence the coupling efficiency of SPP and core guided mode. Figure 4 illustrates the variation of modal loss spectra at different wavelengths for different D values. Figure 4a,b show the variation of spectral loss with respect to D, for Au nanowires and thin film, respectively. Here, for nanowires the separation of the CSC varied from D = 1 µm to 1.2 µm, and for the thin film, the separation is varied from D = 1.3 µm to 1.5 µm. Here, we can clearly observe the blue shift for AuNWs and Au film with maximum modal loss of 34.90 dB/mm and 34.76 dB/mm at D = 1.1 µm and 1.4 µm respectively. In both cases the higher loss for D = 1.1 µm and 1.4 µm may be attributed to the fact that, far from the core of optical fiber, the interaction region of field of the core guided mode with metal–dielectric interface is higher, due to the large value of the evanescent field. Hence, we have chosen these separations as the optimum distance of CSC from the core, for both Au nanowires and thin film.

**Figure 5:**
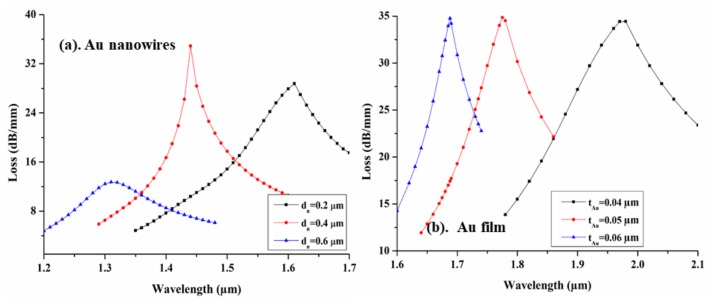
Variation in loss spectra with the wavelength for at different d_n_ and t_Au_ (**a**) AuNWs and (**b**) Au film, respectively.

Figure 5 illustrates the variation in modal loss spectra at various nanowire diameters (d_n_) and film thicknesses (t_Au_) at optimized D. From Figure 5a,b we can clearly observe a blue shift in modal loss spectra at various wavelengths. We observe that the sensing response of the designed sensor is highly influenced by d_n_ and t_Au_, and hence their optimization is an important factor for developing a highly sensitive sensor. Before the optimization of the d_n_ and t_Au_, all other parameters are kept constant at their initial values. The nanowire diameter d_n_ is varied at 0.2 µm, 0.4 µm, and 0.6 µm and achieved a maximum peak loss of 34.90 dB/mm at λ_R_ = 1.44, while in the case of Au film, a maximum modal loss of 34.87 dB/mm is obtained for t_Au_ = 0.05 µm at λ_R_ = 1.775 µm. In both cases, due to the change in the diameter of nanowire and thickness of film, the effective refractive index (n_eff_) of SPP mode varies, which leads to the spectral loss shift towards the blue wavelength.

**Figure 6:**
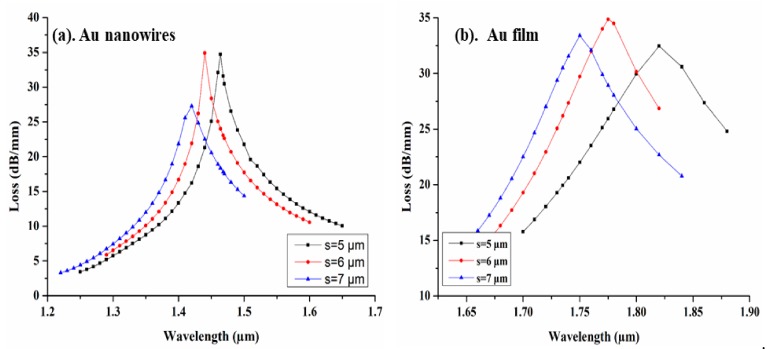
Loss spectra with the variation of channel size for (**a**) AuNWs and (**b**) Au film.

The CSC size (s) also plays an important role in the sensing performance of the proposed design. In order to investigate its influence on the performance of CSRIS, we keep the separation (D) of channel from the core at a fixed distance, while s is varied from 5 µm to 7 µm for both sensors covered with Au film and AuNWs. The modal loss spectra of the fundamental mode with the variation of s is depicted in Figure 6. From Figure 6, we can clearly observe a strong blue shift when the size of channel increases from 5 µm to 7 µm with a maximum modal loss of 34.90 dB/mm and 34.87 dB/mm for AuNWs and film respectively at s = 6 µm.

**Figure 7:**
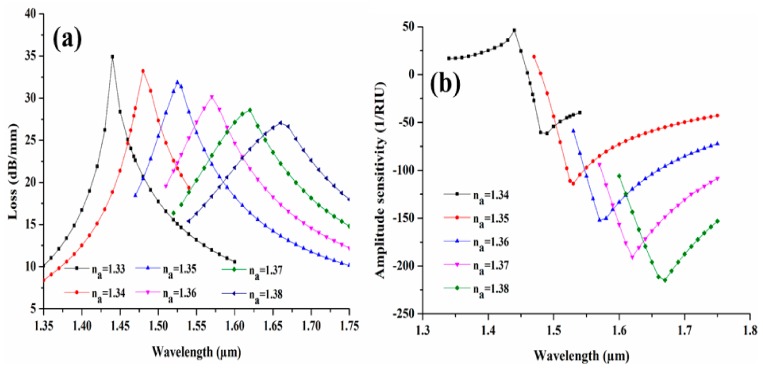
(**a**) Loss spectra and (**b**) amplitude sensitivity due to the changes of n_a_ for AuNWs filled CSRIS.

After optimization of all structural parameters, the designed CSRIS is characterized for a wide range of analytes of varying refractive indices. The variation in modal loss spectra for the CSRIS filled with AuNWs is depicted in Figure 7a. We can clearly observe a good red shift in loss spectra with respect to n_a_ varying from 1.33 to 1.38. Here, we can interpret that the full-width and half-maxima (FWHM) of loss peak broadens with increasing the RI of the analytes. The broadening of these loss peaks occurs due to the comparative lower index contrast because of increase of n_a_. A minimum modal loss depth of 27.07 dB/mm is obtained for n_a_ = 1.38 and the maximum loss depth of 34.90 dB/mm is obtained at n_a_ = 1.33. For some specific wavelengths, the sensitivity of the designed sensor can be measured by using the amplitude modulation technique, which can be defined by the following equation [24]:

(5) $$S_{A}({\mathrm{RIU}}^{-1})=-\frac{1}{\alpha \left(\lambda ,{\;n}_{\alpha }\right)}\frac{\;\partial \alpha \left(\lambda ,{\;n}_{\alpha }\right)}{\partial n_{\alpha }}$$

where, α (λ, n_α_) is defined as the modal loss value for any analytes (n_a_) and ∂α (λ, n_α_) is the modal loss difference of analyte RIs. The amplitude sensitivity graph is illustrated in Figure 7b for n_a_ varying from 1.33 to 1.38.

**Figure 8:**
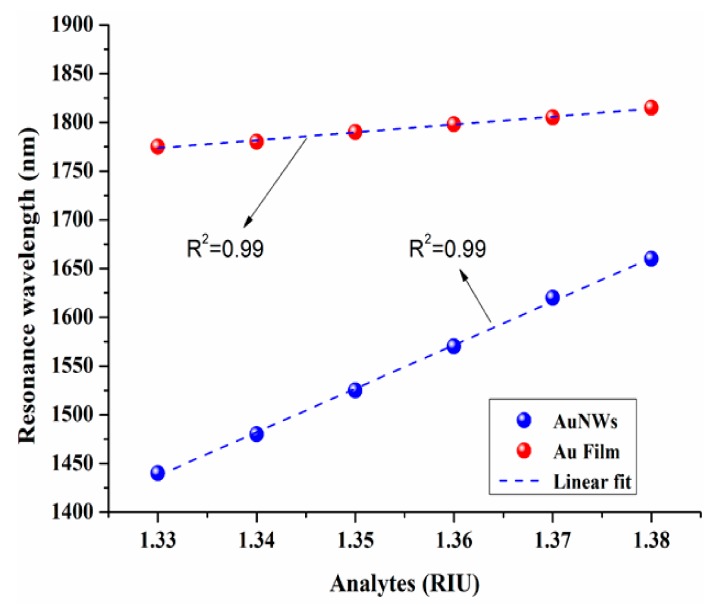
Sensitivity plot of the designed sensors for both AuNWs and Au film.

The performance of proposed CSRIS is investigated in terms of their wavelength sensitivity, amplitude sensitivity, and linearity. The relationship between resonance wavelength and corresponding analytes are shown in Figure 8, for both AuNWs and Au film. From the figure, we can clearly observe a good linearity of R^2^ = 0.99 for the n_a_ varying between 1.33 to 1.38. The wavelength sensitivity (S_λ_) of the designed sensor can be calculated by the following equation [25]:

S_λ_ = Δλ_peak_/Δn_a_ (nm/RIU) (6)

where Δλ_peak_ shows the change in resonance wavelength and Δn_a_ represents the change in analytes refractive index. The CSRIS covered with Au film results in an average sensitivity of 808.57 nm/RIU, which is enhanced five times by using AuNWs in the same CSC and achieve a high sensitivity of 4471 nm/RIU for RI varying between 1.33 and 1.38. In addition to its high sensitivity, the main advantage of the proposed sensor is in its design. Here we have incorporated a CSC just above the core to minimize the wastage of analytes, as it requires only a small amount of measurand to be detected. Moreover, the sensor is easy to fabricate and implement, in contrast to the conventional photonic crystal fiber (PCF) sensor, due to the external metal-coated structures. In the AuNWs, the metal is chemically inert, stable in the long-term, biocompatible, and the nanowires also do not suffer from the oxidation issue. Due to all these features, the proposed sensing device can be used effectively in chemical and biological sensing. Table 2 shows the comparative sensitivity values of various reported articles.

### Fabrication Tolerance

**Figure 9:**
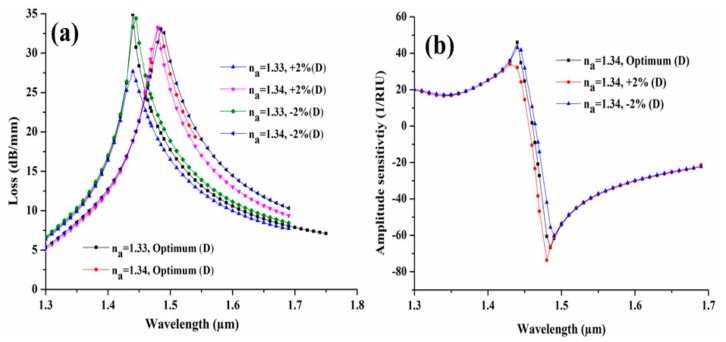
(**a**) Loss spectra and (**b**) amplitude sensitivity, with ±2% variation in separation of CSC from core.

In general, it is very difficult to fabricate a sensor with such exact optimized parameters. Usually, a small variation of ±1% may take place during the fabrication process. To validate the sensing performance of the proposed sensor we have investigated the structural parameter with ±2% fabrication tolerance. Figure 9 exhibits the influence of the CSC separation (D) on modal loss spectra with the variation of ±2% from its optimum value. From Figure 9a, we can clearly observe that the modal loss decreases to 27.68 dB/mm and 34.41 dB/mm from its optimum value of 34.90 dB/mm, due to +2% and −2% variation in D, respectively, at the n_a_ = 1.33. Similarly, for n_a_ = 1.34, the loss decreases to 31.87 dB/mm and increases to 32.98 with +2% and −2% variation in D, respectively, for its optimum value of 33.23 dB/mm. Figure 9b illustrates the amplitude sensitivity corresponding to this variation and it can be clearly seen from the figure that the maximum amplitude sensitivity is increased by 6.95 RIU^−1^ and decreased by 6.58 RIU^−1^ due to ±2% variation of D.

**Figure 10:**
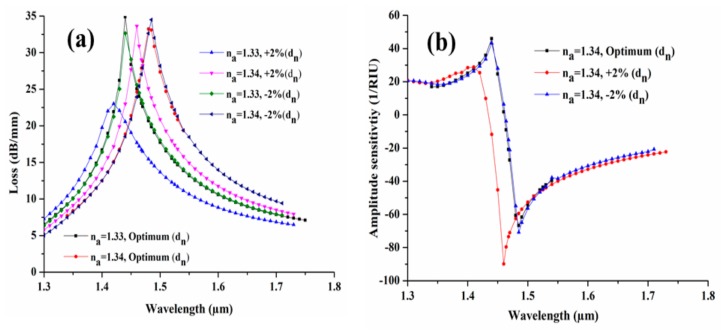
(**a**) Loss spectra and (**b**) amplitude sensitivity, with ±2% variation in d_n_ of AuNWs.

In addition, the ±2% in diameter (d_n_) of AuNWs are shown in Figure 10. From Figure 10a the decrease in loss to 23.01 dB/mm and 32.65 dB/mm is clearly observed with +2% and −2% variation at n_a_ = 1.33 from its optimum value of 34.90 dB/mm. The amplitude sensitivity is also drawn from the obtained spectral loss and shown in Figure 10b. From the figure, we can state that the amplitude sensitivity is increased by 23.23 RIU^−1^ and 4.08 RIU^−1^ with +2 and −2 % variation, while the optimum sensitivity is obtained of 66.70 RIU^−1^.

**Figure 11:**
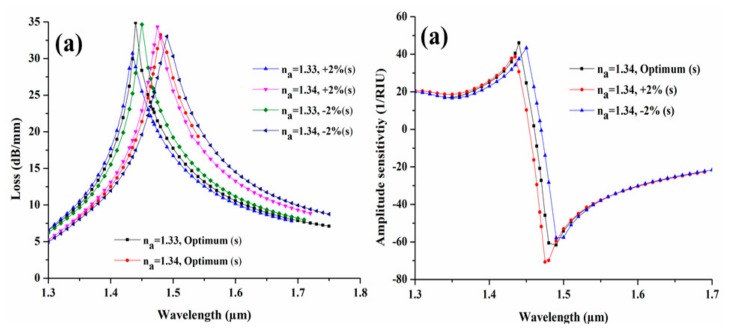
(**a**) Loss spectra and (**b**) amplitude sensitivity, with ±2% tolerance in CSC size (s).

Furthermore, we have also investigated the tolerance response for CSC size (s) with ±2% variation in their optimized parameter. Figure 11 illustrates the influence of variation on its modal loss and the amplitude sensitivity. Figure 11a shows the similar behavior as we have seen in Figure 9. Here, we have obtained that the loss spectra is decreased to 30.17 dB/mm and 34.65 dB/mm from its optimum value 34.90 at n_a_ = 1.33, whereas for n_a_ = 1.34, the spectral loss get increased to 34.34 dB/mm and decreases to 33.02 dB/mm from its optimum value of 33.23 dB/mm for +2% and −2% variation. Figure 11b illustrates the amplitude sensitivity plot for the ±2% variation in channel size and obtained an increase in sensitivity by 3.97 RIU^−1^ for +2 % variation, while it obtained a decrease in sensitivity by 8.86 RIU^−1^ from its optimum sensitivity of 66.70 RIU^−1^. From all the investigations, we may see the negligible variation in sensitivity and the sensing response of the designed sensor with ±2% in their structural parameter. Hence, we can say that our designed sensor can perform effectively for a broad range of applications such as chemical sensing, biosensing, gas sensing, etc.

## 4. Conclusions

In summary, we have designed a new kind of CSRIS for a wide range of RI sensing in this paper. The optimization of structural parameters and the sensing performance of the device are numerically analyzed by using FEM technique based on COMSOL Multiphysics. A comparative analysis is carried out here using thin film and AuNWs in CSC in order to select the best for the sensitivity enhancement of the designed sensor. The simulation results exhibit that concave shaped fiber covered with AuNWs results a high wavelength and amplitude sensitivity of 4471 nm/RIU and 214 RIU^−1^, respectively in comparison to Au film, in the n_a_ range varying between 1.33–1.38. The impact of optimization of the structural parameters are also studied in the present work. From the above study, we can state that the use of AuNWs can effectively enhance the sensitivity of the sensor and the structural parameters such as D, d_n_, and s of CSRIS are the key factors to achieve a high sensitivity. The sensing response of the designed CSRIS shows that the device can be used in various chemical and biological sensing. In addition to the high sensitivity, its design requires only a small amount of measurand for the sensing, which is one of its major advantages over other conventional sensors.

## Figures and Tables

**Table 1:** Optimized structural parameters for the proposed sensor.

Sensing Layer	Distance (D)	Thickness (t_Au_) / Diameter (d_n_)	Channel Size (s)	Analytes (n_a_)
Au film	D = 1.4 µm	t_Au_ = 0.05 µm	s = 6 µm	1.33
AuNWs	D = 1.1 µm	d_n_ = 0.40 µm	s = 6 µm	1.33

**Table 2:** Sensitivity comparison of various reported sensor based on metallic nanowires.

Sensor Design	Sensitivity (nm/RIU)	RI Range
Photonic crystal fiber filled with gold nanowire [26]	2000	1.370–1.450
Grape fruit fiber filled with silver nanowire [27]	2400	1.330–1.335
Extra-broad photonic crystal fiber filled with nanoscale gold wires [28]	3233	1.300–1.790
**Our proposed design with AuNWs**	**4471**	**1.330–1.380**
